# Point-of-care diagnostic devices for periodontitis – current trends and urgent need

**DOI:** 10.1039/d3sd00317e

**Published:** 2024-06-03

**Authors:** Alexandra Griffith, Charmi Chande, Sahitya Kulkarni, Josuel Morel, Yu-Hsuan Cheng, Emi Shimizu, Carla Cugini, Sagnik Basuray, Vivek Kumar

**Affiliations:** a Department of Biomedical Engineering, New Jersey Institute of Technology Newark NJ 07102 USA sbasuray@njit.edu vak@njit.edu; b Department of Chemical and Materials Engineering, New Jersey Institute of Technology Newark NJ 07102 USA; c Department of Biological Sciences, New Jersey Institute of Technology Newark NJ 07102 USA; d Department of Endodontics, Rutgers School of Dental Medicine Newark NJ 07103 USA; e Department of Oral Biology, Rutgers School of Dental Medicine Newark NJ 07103 USA

## Abstract

Point of care (POC) diagnostic devices provide a method for rapid accurate identification of disease through analysis of biologically relevant substances. This review focuses on the utility of POC testing for early detection of periodontitis, a critical factor in treating the disease. Accessing the oral cavity for biological sampling is less invasive when compared to other internal test sites, and oral fluids contain biomarkers indicative of periodontitis. The ease of access makes the mouth an excellent target location for the development of POC devices. In this review, accepted standards in industry by which these devices must adhere, provided by the World Health Organization such as REASSURED and CLIA, are discussed. An overview is provided for many periodontal biomarkers currently being investigated as a means of predicting periodontal disease and its progression. POC devices currently being investigated for the identification and monitoring of periodontal disease such as paper-based and lab-on-a-chip based devices are outlined. Limitations of current POC devices on the market are provided and future directions in leveraging biomarkers as an adjunctive method for oral diagnosis along with AI-based analysis systems are discussed. Here, we present the ESSENCE sensor platform, which combines a porous non-planar electrode with enhanced shear flow to achieve unprecedented sensitivity and selectivity. The combination of the ESENCE chip with an automated platform allows us to meet the WHO's ASSURED criteria. This platform promises to be an exciting POC candidate for early detection of periodontitis.

## Abstract 2

Critical review of point of care devices in oral care.

## Graphical abstract

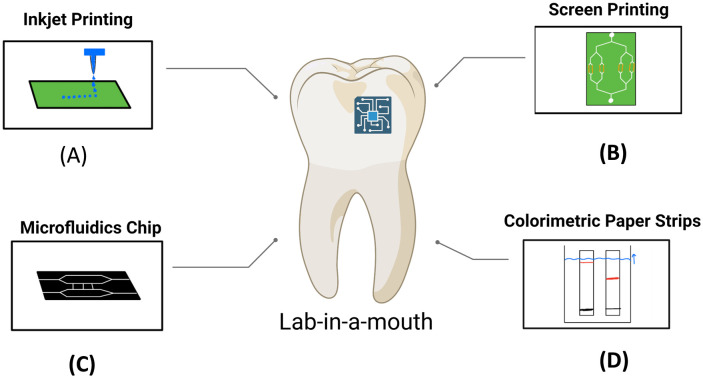

## 1. Introduction

### 1.1 Cost and insurance hurdles

Oral health is vital to people of all ages, including children, adults, old age people, and Medicare beneficiaries with disabilities, and is connected to overall health.^4^ But often, oral health needs to be recognized, and maintenance is challenging due to many factors, including cost and insurance hurdles.^6^ Medicare's national health insurance program does not cover dental care and routine dental checkups. Almost 65%, nearly 37 million people, do not have dental coverage through Medicare. Consequently, the patient does not visit the dentist (almost 49%). Limited dental insurance, like Medicare beneficiaries using dental services (nearly 19%), leads to a high amount of out-of-pocket costs ($1000) for oral health care.^7^ Such a high cost makes dentist visits and dental procedures unaffordable. There are options for dental care advance plans like Medicare Advantage, private dental, Medicaid, and employer-sponsored plans. However, such plans are often provided just to the working age, only cover regular dentist visits, and are often subjected to annual caps.^8^ One must pay out-of-pocket for complicated dental issues. Adults aged more than 60 years require more attention for oral health for an incident; 14% or more adults have untreated caries, and about 68% have periodontitis.^9^ Most adults over 65 who do not have natural teeth are prone to difficulties such as chewing and swallowing, leading to health complications.^9^ Untreated caries, periodontitis, and edentulism are subject to high-cost emergency room visits, forcing them to spend more. Hence, attention is required to deal with such challenges and find a way to make dental care more accessible and affordable. The Centers for Medicare and Medicaid Services (CMS) is reviewing a plan to cover oral health, illnesses, and injuries with Medicare.^10^ Other considerations include a premium-based model like voluntary dental benefits, similar to the Part D drug benefit prescription.^11^ There is a need to propose better solutions for dental health plans since poor oral care results in complicated health risks. Another approach to reduce cost and make oral diagnosis available to a large population of different age groups would be technological development, accessibility, and affordability. Advances in POC diagnosis systems have recently gained immense attention due to advantages like chairside operation, easy operation, and quick analysis protocols.^12^

### 1.2 Summary of oral POC diagnostic device classes

Point of care (POC) diagnostic devices represent a method of clinical monitoring and testing that allows for quick and accurate identification of the medical malady in the office or while the patient is close at hand. POC diagnostic devices can rapidly identify biologically relevant substances like blood glucose levels,^13^ liver enzymes,^14^ blood in the stool,^15^ pregnancy,^16^ and the presence of drugs in the blood or urine amongst others.^17^ Though many varieties of POC devices exist, diagnosis of infectious diseases is of great global interest. The World Health Organization's criteria for an effective POC test for such cases are summarized by the acronym ASSURED; the device must be affordable, sensitive, specific, user-friendly, rapid and robust, equipment-free, and deliverable to end users.^18,19^ This has been updated to account for real-time health monitoring with a revision to the ASSURED criteria, now termed REASSURED, to include real-time connectivity and ease of specimen collection to the initial standards.^19^ The oral cavity is more easily accessible to health care providers in a less invasive manner than the GI tract, vascular system, or other body parts. Rapid and accurate diagnosis of oral pathologies and their pathogenic basis is beneficial to ensure that the most appropriate treatment is used and the illness can be treated quickly. Fluids accessible and assessable in the mouth, like saliva, mouth rinse, peri-implantitis fluid (PISF), and gingival crevicular fluid (GCF), allow noninvasive testing for notable biomarkers relevant to both specific oral diseases like periodontitis and systemic issues.^20^

#### 1.2.1. Consumer-targeted POC devices

Many off-the-shelf POC devices are available over the counter. These products yield easy-to-understand results, allowing patients to monitor their health personally or know when to seek professional care. Perhaps the most common instance of an at-home POC device is the pregnancy test, which reacts to hCG in the urine. Rapid tests for COVID-19 use a similar antibody-based detection system for at-home identification of the illness.

#### 1.2.2. Clinician targeted POC devices

Various rapid diagnostic tests are performed in clinical or hospital settings. These POC tests meet the WHO ASSURED criteria but are not available for use by the general public. Examples include the rapid antigen streptococcal screen (Strep A test), which uses samples collected by swabbing the tonsils to quickly detect the presence of the causative bacterium.

#### 1.2.3. Insurance targeted POC devices

Classical diagnostic imaging and testing in the dental and craniofacial space can be costly. For this reason, a cheaper and more efficient alternative may be required as a preliminary screen to gain insurance approval for a secondary and more expensive technique. For example, an in-office X-ray machine may be used first to indicate the need for a more expensive cone beam CT scan.

### 1.3 Biosensors in the oral cavity

A subset of POC devices used in medicine are biosensors, which generate a measurable signal in proportion to a detected biological reaction or chemical concentration. Every biosensor consists of both static and dynamic elements, which are optimized for increased performance. A biosensor must be selective for the desired analyte, create a reproducible and precise output, have biologically appropriate sensitivity for the minimum and maximum amount of analyte that can be detected, and demonstrate the accuracy of the measured signal; there must be linearity of the measured response.^21^ There has been considerable advancement in the development of biological sensors over the last decade, especially the rise of electrochemical sensors, as they tend to be cheaper to miniaturize than other spectroscopic or chromatographic detection methods.^22^ The combination of next-generation sequencing, salivaomics, and biosensors enables the identification of non-cultivable or difficult-to-culture microbes that were previously undetectable using traditional cultivation methods from oral fluids. A critical property most relevant in the case of dental and craniofacial biosensors is the stability of the device. Biosensors are susceptible to ambient disturbances in the area surrounding the device, which can change the precision and accuracy of the system. Long incubation periods or continuous monitoring increases the likelihood of a stability disturbance.^21^ In the oral cavity, the changes caused by breathing, speaking, mastication, and the introduction of exogenous materials like food, drink, or removable orthodontic systems can cause disturbances that must be accounted for.

**Fig. 1:**
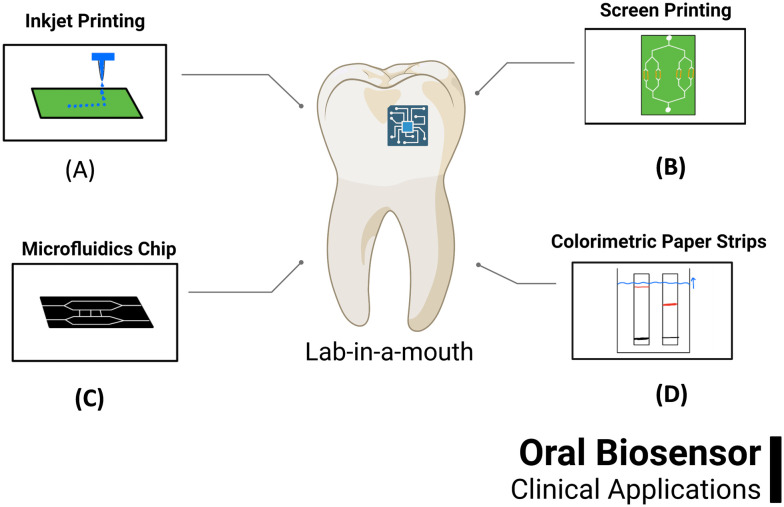
Emerging methods for oral diagnosis by POC: (A) inkjet printing,^1^ (B) screen printing,^2^ (C) microfluidic chip^3^ and (D) colorimetric paper strips.^5^

Despite this, the National Institute of Dental and Craniofacial Research (NIDCR) is especially interested in funding research into the use of biosensors in the oral cavity as the mouth can be conveniently accessed during office visits.^23^ The mouth offers a variety of appropriate biosensor attachment surfaces. Hard tissues like the teeth or bone, firm tissues like the gingiva or hard palate, or soft tissues like the cheeks are all possible placements for short or long-term *in situ* biosensors.^24^ Additionally, as the mouth is the entry point for both the GI tract and the lungs, biosensors placed within it will be in a location that serves as an entry point for bacteria and viruses, medications, toxins, and nutritional substances, information about which could be used in the diagnosis and management of a variety of health conditions. Some of the methods in development for oral diagnosis by point-of-care devices are seen in Fig. 1.

## 2. Requirements

### 2.1 CLIA requirements for POC devices (ASSURED compliant)

In addition to the ASSURED criteria mentioned previously, novel POC devices for implementation in the oral cavity must be CLIA-compliant except under specific circumstances. The Clinical Laboratory Improvement Amendments of 1988 (CLIA) regulations are federal standards that must be met in the testing of human specimens (tissue, saliva, blood, *etc.*) for diagnosis, prevention, or treatment of disease.^25^ CLIA standards protect human patients from potentially unsafe or unreliable testing. For example, faulty cholesterol tests resulting in high cholesterol being reported as normal can devastate an unsuspecting patient.^26^ Laboratories can apply for CLIA certification by filling out form CMS-116 online and sending it to their local state agency for the state where the laboratory is located. Interested programs should also check with their state agency for other state-specific requirements to ensure compliance.^27^

There are also circumstances where a laboratory may be exempt from CLIA testing. Specific tests are CLIA waived because they are determined “to be so simple that there is little risk of error”. These tests include glucose, urine, pregnancy, cholesterol, and fecal occult blood tests.^25^

### 2.2 Clinical care requirements for POC devices

Early detection of periodontal disease by clinicians is imperative for effective treatment and prevention of disease progression. A predictive test for periodontitis is needed while still in the treatable stages *versus* a diagnostic tool to supplement current methods. The conventional clinical index for periodontitis includes bleeding on probing (BOP), probing depth (PD), clinical attachment loss (CAL), and bone loss by X-rays. These clinical markers indicate accumulated damage and severity of periodontal disease. However, periodontal biomarkers are being investigated as an additional method for detecting or predicting periodontal disease activity.^28,29^ Currently, the only biomarker that has shown utility as a predictive marker of early periodontitis is macrophage inflammatory protein-1 alpha (mip1a).^30–33^ An increase in macrophage inflammatory protein-1 alpha and monocyte chemoattractant protein-1 with increasing severity of periodontal disease makes effective biomarkers to determine the disease stage.^31,33^ Additionally, inflammatory markers such as interleukin-1β, interleukin-6, and tumor necrosis factor-α mediate inflammation of gums and tissue injury in periodontitis, making them effective, if not specific, biomarkers for identification of the inflammation associated with periodontal disease.^34^

For example, gingival crevicular fluid (GCF) or saliva can aid clinicians in determining periodontitis or the severity of periodontitis. For example, matrix metalloproteinases, namely MMP-8 and MMP-9, are significantly elevated in the GCF of periodontitis patients.^35–37^ They are markers of collagen degradation, a classic symptom of periodontal disease.^35^ However, it has been shown that it is the active form of MMP-8 (aMMP-8) that is a relevant biomarker for periodontitis and periimplantitis rather than the total or latent proMMP-8.^37^ The active form of MMP-9, or aMMP-9, is also a useful biomarker for the identification of periodontitis in addition to the latent MMP-9 (IMMP-9).^38^ Human odontogenic ameloblast-associated protein, found in gingival crevicular fluid, indicates detachment of the junctional epithelium from the tooth surface in periodontitis, making it a possible biomarker for clinical use.^39^ Cathepsin G is increased in adult periodontitis GCF when compared with healthy controls. This increase reflects the disease process in nearby inflamed gingiva and improved response to microbiota or dental plaque in periodontitis lesions.^40^ In addition, calprotectin levels in gingival crevicular fluid and plasma are elevated in patients with periodontitis compared to healthy controls.^41,42^

Neutrophil elastase (NE), usually localized within the cytoplasm of neutrophils, is increased in the saliva of periodontitis patients and can also be used as a biomarker for disease progression.^43^ The mechanism by which NE is released and its role in disease is currently unclear; future studies on this mechanism may further elucidate the role of NE as a biomarker for periodontitis. Several Gram-negative bacteria are often implicated in periodontal disease, which can provide evidence of disease. Depending on the patient, the relevant species can include *P. gingivalis*, *T. denticola*, *P. intermedia*, and *A. actinomycetemcomitans.*^44–47^ Specifically, the gingipain proteases of *P. gingivalis* were shown to be a promising biomarker for periodontal disease.^48^ Other biomarkers include the presence of pathogen-associated molecular patterns (PAMPs) like lipopolysaccharides (LPSs) that are present in all Gram-negative bacteria.^49,50^ With further testing and development of identification methods, clinicians can use these biomarkers for early detection of periodontal disease, augmenting current treatment and prevention options.

## 3. State-of-the-art oral diagnostics

### 3.1 Clinical practice

Periodontitis is considered one of the most chronic oral diseases. The onset of periodontitis leads to deep pockets between the tooth surface and the gingival epithelium. In clinical practice, probing pocket depth (PPD) is the most widely used method for assessing corono-apical extension and pocket depth.^51^ Williams and CPITN probes traditionally used in clinical settings have complications affecting results, including probing pressure and angulation, the probe material, shape and size, and reference deposition.^52^ Advancements in state-of-the-art technology like the Vivacre TPS probe offer advantages like computer-linked pressure-sensitive electronic or manual probes to measure the pocket depth, tooth mobility, and attachment level with precise control and high reproducibility.^53^

Radiography is another standard method used in clinical practice, essential to display the change of the architecture and the position of alveoli in a periodontal examination. Traditionally, radiography can reveal bone destruction but fails to articulate the degree of destruction for detecting bone demineralization. Additionally, the superposition of images can also distort the accuracy of the results. Panoramic radiography is advantageous for forming a complete planar image of the dentition. However, it fails to provide a complete 3D structure or visualize subtle alveolar changes.^54^ Cone-beam computed tomography systems (CBCT), called dental CT, have advanced traditional diagnostic imaging methods. CBCT is used to examine the depth of alveolar bone for implants with a relatively low radiation dose, and it reflects the actual size of the alveolar osseous without overlap or distortion.^55^ Despite advances in clinical practices for oral diagnosis, there is a need for new technologies to provide data on the real-time progress of diseases. Table 1 provides details of clinical methods with advantages and disadvantages.

**Table 1:** Current clinical methods used by clinicians for analysis and diagnosis of periodontal disease

Clinical methods	Clinical relevance	Advantages	Disadvantages	Ref
Periodontal probing	Degree of gingival bleeding, the extent of soft-tissue destruction by pocket probing depth analysis, attachment level	Easy to perform by the dentist in the hospital or clinical setting	Difficult to operate on-site for analysis	56–59
Radiography	Type and extent of alveolar bone loss and bone-tissue destruction analysis	One of the best methods in a clinical setting	It is high cost and requires a technician	54 and 60
Inspection	Gingival index changes analyzing the color, shape, and degree of recession	It does not require complicated instruments	Prone to human error in analysis	61 and 62
Percussion	Determines the status of the periodontal ligament	It does not require complicated instruments	Indirect test	63–65
Palpation	Tooth mobility changes and texture of the gingiva	It does not require complicated instruments	Requires technical training to perform the analysis	66 and 67

### 3.2 Research and development

The NIDCR invests in salivary diagnostic research because saliva contains many of the same biomarkers as blood while being more accessible to collect. This allows for quick, low-cost, and non-invasive testing that can be repeated easily to monitor health conditions over time. Salivary diagnostic research has the potential to identify biomarkers associated with oral cancers, anti-HIV antibodies, and many more.^68^

Quick detection of biomarkers associated with periodontitis is important, as early symptoms of the disease may be easily missed or overlooked by the patient. Early diagnosis is valuable as treatment after detection can prevent periodontitis from becoming so severe that vital supporting tooth structures are permanently lost. Monitoring the progression of periodontal disease over time is also essential for dentists to stay up to date with the patient's health. Many of the tests required for this level of disease monitoring require either specialized and bulky equipment or that the patient samples must be sent away for testing, meaning results take longer to acquire. Many existing methods used in clinical studies for detecting these critical salivary biomarkers rely on ELISA or immunofluorescence assays, which are not possible to perform chairside while also using expensive equipment and large volumes of reagents.^69^ Current point of care (POC) systems being investigated to identify and monitor periodontal disease are outlined in the following section and Table 2.

**Table 2:** Current point-of-care (POC) systems being investigated for use in the identification and monitoring of periodontal disease subdivided by category, including point-of-care tests (POCTs), paper-based POCT, and lab-on-a-chip (LOC)

Classification	Sample	Detection principle	Biomarker/target analyte	Limit of detection	Ref.
POCT	Saliva	Surface acoustic waves	MMP-8	62.5 ng ml^−1^	80
	Saliva	Surface plasmon resonance (SPR)	MMP-9	8 pg mL^−1^	81
	Commercial saliva & gingival crevicular fluid (GCF)	Colorimetric	Human neutrophil elastase & cathepsin-G	1 pg mL^−1^	82
	Saliva	Fluorescence resonance energy transfer (FRET) and SPR	Odontogenic ameloblast-associated protein	1.63 nM	83
					84
	Saliva & GCF	Visual detection	Gingipain protease	49 CFU mL^−1^	48
			(*Porphyromonas gingivali)*		
	Saliva	Immunoassay	IL-1β	28 pg ml^−1^	85
			IL-6	5.5 pg ml^−1^	
			MIP -1α	5 pg ml^−1^	
			MMP-8	165.9 ng ml^−1^	
	Saliva	Electrochemical	Human odontogenic ameloblast-associated protein (ODAM)	1 nM	79
	Saliva	Surface plasmon resonance (SPR)	MMP-8	225 pM	86
Paper-based POCT	Subgingival plaque	Immunochromatography	40 k-outer membrane protein of *P. gingivalis*	5 copies per mL	87
	Saliva	Immunochromatography	Monoclonal antibodies of *P. gingivalis*	10^5^ cells per ml	88
	GCF	Immunochromatography	MMP-8	1 mg L^−1^	89
	Saliva	Colorimetric	Nitrite	10 μM L^−1^	90
	Saliva	Immunochromatography	MMP-8	25 ng ml^−1^	91
			MMP-9	100 ng ml^−1^	
	Saliva	Proteolytic activity	Cathepsin-G	100 fg ml^−1^	92
		(Visual detection)	Neutrophil elastase	1 pg ml^−1^	
	Saliva	Proteolytic activity	Gingipains of *P. gingivalis*	49 CFU per ml	93
		(Visual detection)			
	Mouth rinse	Immunochromatography	aMMP-8	25 ng ml^−1^	94
	GCF	Disk-like lateral flow immunoassay	MMP-8	5.455 ng ml^−1^	28
			IL-1β	0.054 ng ml^−1^	
			TNF-α	4.439 ng ml^−1^	
	Gingival crevicular fluid	Isothermal recombinase polymerase amplification + lateral flow strips	*P. gingivalis*	9.27 CFU per reaction	75
LOC	GCF	Current continuous flow polymerase chain reaction (CF-PCR)	*Treponema denticola*	125 CFU per μl	77
	GCF	Sandwich enzyme-linked immunosorbent assay	Calprotectin	1.56 ng ml^−1^	95
	Bacterial culture	PCR	*P. intermedia*	2000 copies per reaction	96
			*A. actinomycetemcomitans*	10 copies pe reaction	
			*P. gingivalis*	10 copies per reaction	
			*T. denticola*	20 000 copies per reaction	
Electrochemical sensing	Saliva	Electrochemical	Salivary peroxidase	0.5 ng ml^−1^	97
	Artificial saliva	Electrochemical	Antimicrobial peptides (magainin I)	10^2^ CFU per mL	84
			(*Streptococcus sanguinis)*		
	Saliva	Electrochemical	IL-1β	0.014 ng ml^−1^	78
			MMP-8	0.13 ng ml^−1^	
	Saliva	Electrochemical	Human odontogenic ameloblast-associated protein (ODAM)	1 nM	79

#### 3.2.1. POCT – point of care testing

A variety of point-of-care tests (POCTs) for use in the diagnosis and monitoring of periodontitis have been explored using several detection principles, including surface plasmon resonance (SPR), surface acoustic waves, colorimetric methods, and fluorescence resonance energy transfer (FRET). Other devices include the use of novel molecularly imprinted polymers (MIPs) for on-site detection of oral diseases.^70^ Wearable mouthguards have played a crucial role in the diagnosis and monitoring of oral health. They enable the assessment of various biochemical parameters, such as monitoring saliva turbidity to assess oral hygiene and conducting saliva glucose monitoring, among other functions, providing real-time data.^71^ Some of the most recent advances in the area use electrochemical sensors to make portable and rapid devices for the chairside detection of biomarkers associated with periodontal disease. Using these electrochemical sensors allows for higher sensitivity and selectivity in initial diagnosis, aiding in the reduction of false positives and negatives and allowing for more accurate and precise POCT systems for the detection of periodontal disease progression. In addition, the use of paper-based POCT systems allows for clinicians to receive diagnostic results easily and accurately when compared to current diagnostic methods.

##### 3.2.1.1. Paper-based POCT – paper-based point of care testing

**Fig. 2:**
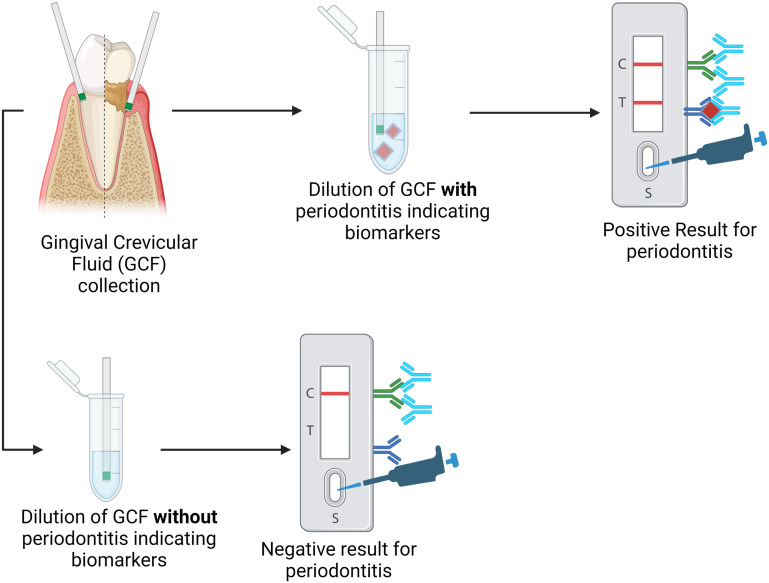
Schematic for the process of the qualitative paper-based lateral flow immunoassay for diagnosis of periodontal disease based on the presence of multiple biomarkers.

Many paper-based POCTs are being developed as the paper-based test strip requires even less equipment when used chairside.^72^ Detection methods are more limited when the POCT is paper-based, typically relying on immunochromatography or colorimetric readouts, which are detected visually, making them less quantifiable than electrochemical results. Although the results from paper-based POCT systems may have less sensitivity than electrochemical POCT or other methods when used chairside, they give dentists and physicians a reliable, cost-effective, simple, and time-efficient approach to the diagnosis and disease tracking of periodontitis.^73^ A workflow for conventional paper-based POCT used in detecting and measuring periodontitis biomarkers is seen in Fig. 2 based on the work done by He *et al.*^28^

The work by He *et al.* used a paper-based POCT to detect periodontitis by developing a disk-like lateral flow immunoassay to simultaneously detect three different biomarkers.^28^ The luminescence probe was able to detect MMP-8, IL-1β, and TNF-α using gingival crevicular fluid in a 30-minute timeframe. The results from this paper-based device also showed high correlation rates compared to standard clinical results. This indicates that the paper-based POCT device shows promise as an accurate and timely way to diagnose and check the progression of periodontitis based on multiple biomarkers.^28^ Using GCF rather than saliva helps to indicate the condition of specific teeth instead of the entire mouth.^74^

Paper-based lateral flow strips (LFSs) can also detect the presence of specific bacteria associated with periodontal disease. Ge *et al.* used isothermal recombinase polymerase amplification (RPA) for nucleic acid amplification paired with a nanoparticle-based lateral flow strip to allow visualization of the results. *Porphyromonas gingivalis*, a bacterial species most closely associated with severe periodontal disease, was detected using species-specific 16S rRNA as the RPA target. Gingival crevicular fluid was used as the analyte, and the LFA test accurately determined the presence of *P. gingivalis,* having results consistent with the more involved and expensive PCR assay used conventionally.^75^

##### 3.2.1.2. LOC – lab-on-a-chip

With many laboratory testing methods requiring extensive and expensive equipment to analyze biological samples, there is a push to miniaturize testing infrastructure and create lab-on-a-chip (LOC) technologies. LOC systems reduce the amount of analyte needed and aim to have high throughput, with rapid analysis time, while being portable and automated.

In diagnosing and monitoring the state of periodontal disease, identifying oral pathogens responsible for the destruction of periodontal tissue may be beneficial.^76^ Identifying the pathogens by culturing the patient samples is typically time-consuming, and a clinical office must be equipped for such a process. Identifying the samples using PCR is a more realistic alternative. Still, the equipment needed for every step in the continuous flow polymerase chain reaction (CF-PCR) process would only be found in laboratories. In addition, many versions of PCR used today have issues concerning long run times, machine size, false positives, *etc.* These issues make PCR a process typically performed in laboratories, preventing its use in POC systems. Li *et al.* successfully created a LOC capable of CF-PCR for identifying key periodontal pathogens: *Porphyromonas gingivalis*, *Treponema denticola*, and *Tannerella forsythia.* To generate a LOC for PCR, this group incorporated automatic sample injection, which removes the need for a more costly syringe pump. This device also achieved rapid DNA amplification and detected the PCR product in sequence without the need for external technology for final confirmation of the presence of the pathogenic bacteria. This LOC all-in-one system lowered the cost of such systems, a significant improvement necessary for implementing such a system in the field today.^77^

##### 3.2.1.3. Electrochemical sensing methods

**Fig. 3:**
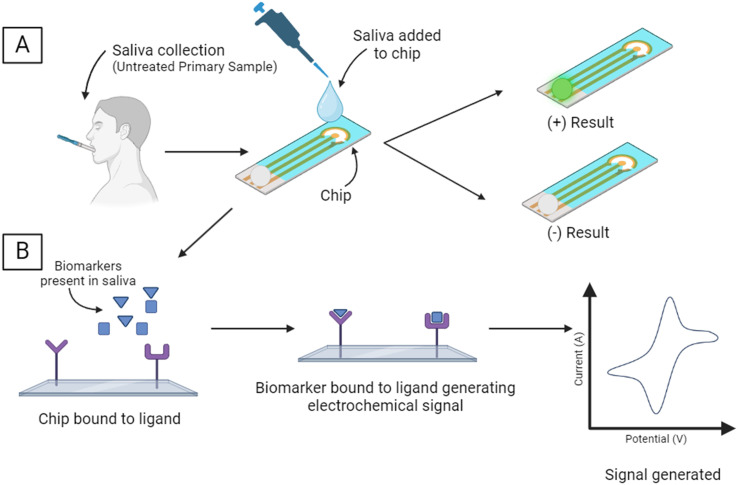
Schematic for the electrochemical biomarker sensing process for the detection of common periodontal biomarkers. [A] Saliva is collected from the patient and added to the device/chip which will yield either a positive or negative result depending on the presence of the biomarker. [B] An electrical signal is generated by the binding of the biomarker to the ligand. Quantification of the biomarkers can be interpreted using the intensity of the generated signal.

Currently, the electrochemical sensing methods for periodontal disease markers are large and costly and cannot be performed chairside and in a cost-efficient manner. Zhang *et al.* have created a small device for the electrochemical sensing of multiple periodontal disease biomarkers. In addition, it has been found that miniaturization of the device made by Joe *et al.* would be valuable to the POCT market. A POCT workflow and mechanism for detecting and measuring periodontitis biomarkers based on the devices made by Zhang *et al.* and Joe *et al.* are seen in Fig. 3.^78,79^

Zhang *et al.* have developed a dual-channel electrochemical immunosensor capable of detecting and measuring the presence of both IL-1β and MMP-8 in saliva as a POCT to aid in the rapid and accurate diagnosis of periodontitis.^78^ The simultaneous determination of the levels of these biomarkers synchronously allows for higher accuracy in diagnosing the current stage of periodontitis.^78^ Tested against both spiked artificial saliva and clinical samples, the device has high sensitivity and a wide linear range using IrO_*x*_/Ti_3_C_2_T_*x*_ nanocomposites. The use of an electrochemical immunosensor allows for the detection process to be more cost-effective, timely, and more accessible to perform than currently used techniques such as immunofluorescence and enzyme-linked immunosorbent assays. Both techniques, while accurate, are costly and require large machinery, which is not practical for chairside diagnosis and monitoring of periodontal disease. The electrochemical sensor produced by Zhang *et al.* shows promise in detecting the presence of the biomarkers mentioned above while being practical for chairside use.^78^

Another electrochemical sensor for the detection of periodontal diseases was developed by Joe *et al.*^79^ This POCT was first validated by the detection of known protein biomarkers for other diseases (cardiovascular diseases and type 2 diabetes), and it was then modified for use in an aptamer-based sandwich-type electrochemical biosensor capable of detecting human odontogenic ameloblast-associated protein (ODAM) in saliva. The binding of the target biomarker to the aptamer creates a redox reaction in the bottom plate of the electrochemical sensor which then attracts a secondary aptamer, producing the resulting positive signal. Miniaturization of the detection equipment is vital in ensuring that the device is usable at the chairside and replacing larger equipment that takes up valuable clinical benchtop space.^79^

## 4. Gaps in the field

### 4.1 Microbiological profile

The oral microbiome consists of around 1000 species of microorganisms, including bacteria (Phyla – *Actinobacteria, Firmicutes, Proteobacteria, Fusobacteria,* and *Spirochetes*), fungi (*Candida, Saccharomyces, Aspergillus, Cladosporium, Fusarium* and *Cryptococcus*) and protozoa (*Entamoeba gingivalis* and *Trichomonas tenax*).^98^ The oral microbiome also accommodates protein signals, the extracellular matrix, enzymatic products, and molecular communications, creating mucosal barriers in the mouth to resist pathogenic invaders.^99^ Viruses, mainly bacteriophages, are found to be associated with the oral microbiome, acting as commensals and pathogens. The bacteriophages release lytic enzymes, altering the composition of the oral microbiome.^100^ The presence of *Aggregatibacter actinomycetemcomitans* was found to be associated with localized aggressive periodontitis.^101^ Extending to the COVID-19 pandemic, viruses could be present in the salivary glands, resulting in asymptomatic carriers.^102^ The oral surface cavity consists of teeth, keratinized epithelium, and nonkeratinized epithelium, making up to 20%, 50%, and 30% of the total surface area, and the mouth cavity consists of the tongue, inner cheeks, and hard and soft palates. Each surface has microbiota and microenvironments, including saliva and gingival cervices.^103^ Disruption of relative proportions of the oral microbiome and the host cells can lead to dysbiosis, which is periodontal disease. Factors causing dysbiosis include diseases such as diabetes, genetic differences, innate/adaptive immune factors, oral hygiene, diet, smoking, the activity of salivary proteins, smoking, and antibiotics/antimicrobial agents.^104^ Some of the key pathogens associated with periodontitis are classified in Table 3 with different color schemes based on the study by Socransky and Haffajee.^105,106^ According to the color scheme, the purple, yellow, and green colors are associated with the bacteria corresponding to periodontal health, and bacteria in orange, red, and other groups are associated with suspected periodontopathogens.^105^

**Table 3:** Socransky color code complexes^105^

Bacteria species	Color complex	Pathogenesis
*Actinomyces*	Purple	Healthy
*Veillonella*		
*Streptococcus: gordonii, intermedius, mitis, sanguis*	Yellow	Healthy
*Capnocytophaga, E. corrodens*	Green	Healthy
*Campylobacter rectus*	Orange	Pathogenic
*Fusobacterium nucleatum*		
*P. micros*		
*P. intermedia*		
*T. forsythia*	Red	Pathogenic
*P. gingivalis*		
*T. denticola*		
*A. actinomycetemcomitans*	Not grouped	Pathogenic
*Selenomonas*		

Non-oral bacteria such as Gram-negative enteric rods, enterococci, and staphylococci are non-resident to the oral microbiome and lead to pathogenesis in mostly non-healthy patients.^107^ The route of introduction could be from the mouth by water, food, chewing items, and many more. Non-oral microbes such as *Acinetobacter baumannii* and *Pseudomonas aeruginosa* are reported as the most pathogenic species causing symptoms leading to periodontitis like periodontal attachment loss.^108,109^ Since the oral cavity habitats have diverse microenvironments comprising highly aerated aerobic microbes and sheltered anaerobic microbes, they represent one of the most complex microbial communities in the human body and are an essential site for diagnosis. With the advance in molecular and point-of-care techniques, oral sampling and analysis are key to diagnosis.^110^ Mitsakakis *et al.* developed a centrifugal microfluidic-based fully automated chair-side POC tool for diagnosing antibiotic-resistant species for oral infections.^111^ Other non-culturing tools explored for the rapid diagnosis of oral microbiome include droplet microfluidics.^112^ However, despite culturing and non-culturing techniques developed for the detection of the oral microbiome, there is high potential in the development of technology establishing rapid chairside detection tactics.

### 4.2 Cost effectiveness

The adoption of point-of-care (POC) technology in the diagnosis of periodontal disease may depend on its cost effectiveness. Existing POC tests for periodontitis like Periosafe, an aMMP-8 point-of-care diagnostic test, have shown improved efficacy in diagnosis of subclinical periodontal disease compared to the conventional bleeding-on-probing assessments.^113^ For the patient, the greatest costs associated with periodontitis occur in the later stages of the disease.^114^ Early identification and treatment result in more predictable treatment outcomes for patients.^115^ A proactive approach, meaning early identification of periodontitis, will result in more savings in the long run.^116^ However, the cost benefits of early identification by POC technology are only realized when the identification is followed by treatment to prevent the expensive later stages of the disease from occurring.^114^ Ultimately, diagnosis alone may not translate into cost effectiveness for patients. For example, analysis of the cost effectiveness of AI screening tools in medicine have shown that while diagnosis by AI may be accurate, its use may not result in cheaper care for the patient.^117^

### 4.3 AI advances

Artificial intelligence (AI) could be defined as machines reducing human efforts and performing human tasks. Machine learning (ML) is a subpart of AI that learns different statistical patterns and predicts unseen data. Both AI and ML have been explored for dental theranostics. Some of the significant advantages of AI include (1) heterogeneous data being generated in dentistry, and AI allows the integration of all the data domains, for example, clinical data, dental history, and biomolecular data, allowing interaction and integration of all the data sets in one place.^118^ (2) Image analysis is routinely carried out in dentistry. AI could drive the analysis of such images by reducing variability in the subjective individual examination, making the entire process error-free and effective, and lowering the entire theranostics costs.^119^ (3) AI allows experimentation with the data sets, which is impossible to perform in real-life settings.^120^ (4) AI could be utilized for record keeping, leveraging speech, voice, and text recognition elements, reducing space and time for data records.^121^ (5) Patients could directly use AI as a promising platform to make the entire theranostic process personalized by collecting data from wearable devices or POC devices. (6) AI could overcome on–off-medicine, where patients barely visit doctors in a real-life setting due to many factors like high out-of-pocket costs, which could be minimized by continuous non-invasive monitoring.^122^ Periodontitis constitutes a significant cause of tooth loss which is called periodontally compromised teeth (PCT).^123^ Despite advancements in the diagnosis and treatment of PCT, real-time prediction of the stage of PCT is not yet possible.^124^ Lee *et al.* used AI and deep learning to evaluate the prediction and diagnosis of PCT. The deep learning approach accurately determined the need for tooth extraction at 73.4–82.8%.^125^ Though AI has numerous advantages, some disadvantages include the possibility of bias and data security breaches, transparency of the AI algorithm, and sometimes higher costs of the AI software.^126^ A mixed approach is needed in the future, where AI could be integrated from multiple data sets from dentistry to make it accessible to a large population.

## 5. Portable platform

### 5.1 ASSURE criteria

POC devices in the market suffer from two significant limitations: 1) false-negative test due to the low number of species of interest present in the sample matrix degrading sensitivity. 2) The sample matrix may contain other molecules similar in functionality to the target molecules, contaminating the sample and affecting selectivity. Additionally, in the case of POC diagnostic devices, the design must facilitate rapid analysis without using expensive or bulky equipment. Sensitivity could be increased by additional, costly steps such as amplifying the analyte, concentrating the analyte, and using exogenous labels in combination with optical or electrochemical systems to amplify the detection signals. Selectivity could be mitigated by additional steps such as extensive purification steps like passing through different resins and isolating the species of interest for diagnosis. Multiplexing is challenging in POC; the current method requires various sensors/protocols to detect other classes of molecules. The current state-of-the-art methods like ELISA (enzyme-linked immunosorbent assay) and paper-based assays are plugged with bulky and expensive instruments or high-cost additional optical image analysis systems, making them relatively costly. Thus, the state-of-the-art sensors often do not meet the “ASSURED” criteria for POC diagnostics (Table 4).

**Table 4:** The WHO criteria for POC devices and the ESSENCE device

WHO criteria^18,19^		ESSENCE meeting the WHO criteria^127^
A	Affordable	Channels from double-sided tape, low sample volume ∼80 μl, cheap electrode material
S	Sensitive, no false negatives	Ionic flux confined to nanodomains -> removal of parasitic electrochemical signals
S	Specific, no false positives	Shear-enhanced flow -> removal of non-specific adsorption, biofouling
U	User-friendly	No user inputs, automated fluidics, automated signal collection and analysis
R	Rapid	Detection in 35 minutes
E	Equipment-free	Portable, cheap spectroscopic station
D	Delivered	Regenerative and robust sensor

### 5.2 ESSENCE platform

**Fig. 4:**
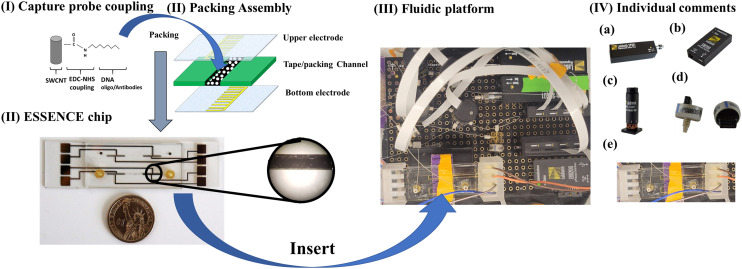
The procedural flowchart encompassing key steps: (I) surface modification of single-walled carbon nanotubes (SWCNTs) *via* an EDC-NHS coupling reaction with an ssDNA probe. These modified SWCNTs are combined with 1X PBS as the packing solution and assembled into the ESSENCE microfluidic chip. (II) A closer look at the ESSENCE chip structure reveals the packed solution electrode on the right-hand side. The microfluidic channel traverses between two electrodes, with the left-hand side electrode as a counter electrode devoid of any packed SWCNT material. A microscopy image highlights the uniform packing on the ESSENCE chip after the packing solution has evaporated from the channel. (III) Integration of the ESSENCE chip into the fluidic platform for automated control. (IV) The components of the fluidic platform can be itemized as follows: (a) a syringe pump that furnishes pressure-driven force within the system, (b) controlling units connected to a laptop for system management, (c) a valve system responsible for regulating sample and buffer solutions, as well as overseeing the overall cleaning process, (d) pressure sensors that provide crucial information about the fluidic conditions, and (e) an installation socket designed to accommodate the ESSENCE chip.

ESSENCE is an electrochemical sensing method that uses a shear-enhanced, flow-through nanoporous capacitive electrode microfluidic analysis system developed by Sagnik *et al.* The ESSENCE chip consists of a flow-through sampling channel sandwiched between two interdigitated microelectrodes (10 μm wide × 500 μm long) (Fig. 4).

The channel is filled with a carbon-based transducer material (CBTM) equipped with a molecular capture agent (antibody, DNA aptamer) specific to the bio-target. When a fluid with the target of interest flows through the cell, the microelectrode signal is attenuated. The CBTM acts as a transducer in the electrochemical cell when a binding event occurs at the CBTM surface. The bio-target is detected using electrochemical impedance spectroscopy (EIS). ESSENCE meets the ASSURED criteria set by the WHO (Table 4).

Most peer-reviewed scientific research on detecting periodontitis focuses on fundamental studies, such as biochemical or surface metrology modifications,^128,129^ aimed at enhancing or synthesizing highly selective and sensitive probe materials. These studies provide several strategies to improve electrochemical signals. On the other hand, ESSENCE offers several notable advantages to support the current generation of electrochemical biosensor studies. The platform primarily focuses on enhancing adaptation technologies, leveraging physical advantages to enhance electrochemical signals. This is achieved by transitioning from the traditional planar electrode interface to a 3-dimensional electrode structure. This modification allows the system to be easily adapted to many current probe studies, offering several benefits through this simple yet crucial tweak. First, its electrode nanoporosity formed by probe materials effectively generates significant shear forces akin to hydrogen bond strength, greatly enhancing selectivity by reducing non-specific adsorption. Secondly, the interdigitated, nanoporous microelectrode design leads to a high signal-to-noise ratio (SNR) achieved through heightened sensitivity and specificity. The nanostructured electrodes confine the ionic flux to nanodomains, resulting in greater sensitivity and an amplified signal, thanks to nanoconfinement effects. Thirdly, the nanoporous electrode architecture promotes the convective transport of the target analyte to the sensing element, overcoming diffusion limitations and reducing assay times. Furthermore, the enhanced convection-mediated transport minimizes signal artifacts, such as parasitic double-layer capacitance, thereby facilitating rapid, high-resolution characterization of binding signals with significantly reduced noise. Lastly, the modularity of ESSENCE enables precise control of shear force through the flow rate, allowing for independent sensitivity and device selectivity adjustment. This decoupling approach helps mitigate issues like biofouling and false positives, which are common challenges in existing technologies, thereby increasing overall efficiency. The flexibility of ESSENCE allows for the utilization of various analytes (*e.g.*, blood, urine, serum) with any capture moiety (aptamer, antibody, oligo) targeting a wide range of molecules (prion, mRNA, DNA, or antigen) for diagnosing oral diseases.

In the case of DNA detection, by only using the most straightforward EDAC reaction on a functionalized carbon nanotube surface, the protocol can be reduced to below 15 minutes, including the priming, washing, and rinsing sub-processes, and still reaches fM LOD. These processes utilize microfluidic technologies to manipulate the fluidic algorithm to increase sensitivity and selectivity for an electrochemical sensing process. LabVIEW/python codes control all the procedures on the ESSENCE platform. These include analyte loading, post-washing, EIS analyzer measuring, and buffer washing procedures. Thus, the ESSENCE platform is a fully automatic “one-stop” sensor platform. It significantly reduces the unreliability of human error and removes the requirements of technician training, making it user-friendly.

## 6. Conclusions and future directions

Future developments in POC-based oral diagnostics will pave the way to new technologies like microfluidics, paper-based analytical devices, the ESSENCE platform, and many more. However, reaching practitioners and convincing them to use new technologies is still challenging compared to their daily clinical practices. Salivary-based biomarkers are highly used for specific and sensitive detection of periodontitis and other oral diseases. However, since there is a need for universal methods for sample collection, like saliva, this leads to variations in the analysis. This must be fixed before incorporating current POC devices in clinical settings. Biomarkers can be used as an adjunctive method for oral diagnosis along with standard clinical practices. Additionally, biomarker-based tests could be combined with an AI-based analysis system to interpret the results efficiently, reducing human-based error.

## Data availability

All data created or analyzed in this study are available on request from the corresponding author.

## Conflicts of interest

There are no conflicts to declare.
